# Epileptogenesis in meningioma: Theories, putative biomarkers, and postoperative risk

**DOI:** 10.1111/epi.18559

**Published:** 2025-07-29

**Authors:** William H. Cook, Conor S. Gillespie, Ali Bakhsh, Anthony G. Marson, Michael D. Jenkinson, Adel E. Helmy

**Affiliations:** ^1^ Division of Neurosurgery University of Cambridge Cambridge UK; ^2^ Institute of Systems, Molecular and Integrative Biology University of Liverpool Liverpool UK; ^3^ Department of Neurosurgery The Walton Centre NHS Foundation Trust Liverpool UK; ^4^ Department of Neurology The Walton Centre NHS Foundation Trust Liverpool UK

**Keywords:** antiepileptic drugs, brain tumor, epilepsy, inflammation, seizure

## Abstract

Cranial meningioma are the most common type of primary brain tumor, and focal onset, tumor‐related seizures affect a significant proportion of patients. Seizures affect 30% of symptomatic preoperative patients and a further 12% of postoperative patients. Although most patients may be cured of their oncological disease by surgery, seizures confer disability, reduced quality‐of‐life, delayed return to driving and work, and increase the risk of sudden death. Tumor‐associated seizures are also more likely to be resistant to antiseizure medications (ASMs). ASMs are limited to treating the symptoms of epilepsy—seizures—but have no disease‐modifying effect on the mechanisms that cause or maintain seizure susceptibility. There is a need to be able to predict who is at risk of developing postoperative seizures for targeted prevention or closer monitoring of those at greater risk. Mechanisms underpinning brain tumor–related seizures are most likely multifactorial and related to morphological, biochemical, and metabolic causes. Brain tumors likely cause cortical hyperexcitability due to irritation caused by mass effect, brain invasion, and peritumoral brain edema. Inflammatory mediators are involved in epileptogenesis in animal models and human seizure syndromes and there are experimental data to support the development of inflammatory mediators as biomarkers for epileptogenesis. Meningioma‐associated seizures are incompletely understood and consequently unpredictable with current knowledge. In this review, we discuss the proposed mechanisms of epileptogenesis in brain tumors and putative neuroinflammatory mechanisms for meningioma‐associated seizures. Ultimately, we evaluate the potential of neuroinflammatory biomarkers of epileptogenesis in meningioma and the current challenges with extrapolating from current literature, which primarily consider epilepsy and intrinsic brain tumors. A prospective randomized controlled trial (STOP'EM: ISRCTN14381346) is open in the UK and will determine the role of two weeks of prophylactic levetiracetam in seizure‐naïve patients undergoing meningioma surgery and provide an opportunity to obtain serial blood measurements from patients to assist with biomarker discovery.

**Key points**

- Meningioma‐related seizures confer disability and reduced quality‐of‐life, and are more likely to be resistant to antiseizure medications.
- Seizure mechanisms are most likely multifactorial and related to morphological, biochemical, and metabolic causes.
- Meningioma likely cause cortical hyperexcitability due to irritation caused by mass effect, brain invasion, and peritumoral brain edema.
- Inflammatory mediators are involved in epileptogenesis in animal models and human seizure syndromes.
- There are experimental data to support the development of inflammatory mediators as biomarkers for epileptogenesis.

## 1 INTRODUCTION

Cranial meningioma are the most common type of primary brain tumor, accounting for 40% of all intracranial tumors.^1^ Meningioma are more common in women, older individuals, and ethnic minority populations including Black and Pacific peoples.^2^, ^3^ Meningioma are classified according to World Health Organization (WHO) 2021 guidelines into Grades 1, 2, or 3 based on histopathological and molecular information.^4^

Focal onset, tumor‐related seizures are a particular problem in meningioma and are present in 30% of symptomatic patients and occur in a further 12% after surgery to remove their tumor.^5^ Early postoperative seizures within the first week are considered provoked, whereas late seizures from postoperative Day 8 are considered unprovoked and if recurrent, are termed epilepsy,^6^ however those with provoked seizures are at greater risk of unprovoked seizures (epilepsy).^7^ Although seizures also occur in many other brain tumors, their association with meningioma can be especially troublesome because many patients will be cured of their tumor but may still be debilitated by having at least one seizure. Seizures have short‐ and long‐term consequences on quality of life, including increased length of hospital stay, delayed neurological recovery, delayed return to driving, delayed return to work, and sudden death.^8^, ^9^, ^10^

Seizures associated with a brain lesion, such as meningioma, are more difficult to treat, and more than a third of those with meningioma‐associated epilepsy will continue to have seizures despite optimum treatment with antiseizure medications (ASMs).^5^, ^11^, ^12^ Despite the development of more than 20 new ASMs over the past three to four decades, there has been no reduction in the 30%–40% of patients with treatment‐refractory epilepsy.^13^ A fundamental limitation is that ASMs treat the symptom of epilepsy, namely seizures, but have no impact on the underlying mechanisms that either cause or maintain a susceptibility to seizures.^14^ Meningioma could be an ideal exemplar to explore those mechanisms and provide insights for the development of new treatments for meningioma‐associated epilepsy and for focal epilepsy in general. Although other entities such as low grade intrinsic tumors have a higher absolute seizure risk,^15^ the prevalence of meningioma makes them a greater health care burden. We therefore believe this is an important issue to address.

Seizure‐naïve patients undergoing meningioma resection are at risk of developing postoperative seizures. There is a need to be able to predict who is at high or low risk for developing seizures. The ability to more accurately predict the risk of seizures in patients with meningioma would help patients and clinicians and may enable the targeted prevention or closer monitoring of those at greater risk. A prospective randomized controlled trial (STOP'EM: ISRCTN14381346),^16^ is open in the UK and will determine the role of two weeks of prophylactic levetiracetam in seizure‐naïve patients undergoing meningioma resection.

The aim of this review is to describe the clinical risk factors of meningioma‐associated seizures, describe proposed mechanisms for seizure occurrence with a focus on inflammation, describe putative biomarkers, and explore the future directions of this field.

## 2 KNOWN CLINICAL ASSOCIATIONS WITH SEIZURES

Multiple studies have attempted to predict meningioma‐associated seizures based on routinely available clinical data but are generally retrospective and affected by selection bias. Tumor factors include edema, temporal location, tumor size, and neurological deficit.^17^ Although intuitive, preoperative seizures also seem to increase the risk of postoperative seizures, and some patients are cured of their seizures following an operation.^17^ Preoperative seizures likely disrupt the peritumoral brain's molecular and enzymatic homeostasis, which persists postoperatively and leaves patients prone to seizures even after the tumor is removed.^18^ Although many studies have related peritumoral edema to pre‐ and postoperative seizures,^19^, ^20^, ^21^ meta‐analyses have demonstrated conflicting results.^5^, ^22^ Therefore, edema most likely contributes in concert with other unconfirmed factors to increase the risk of seizures.

**FIGURE 1:**
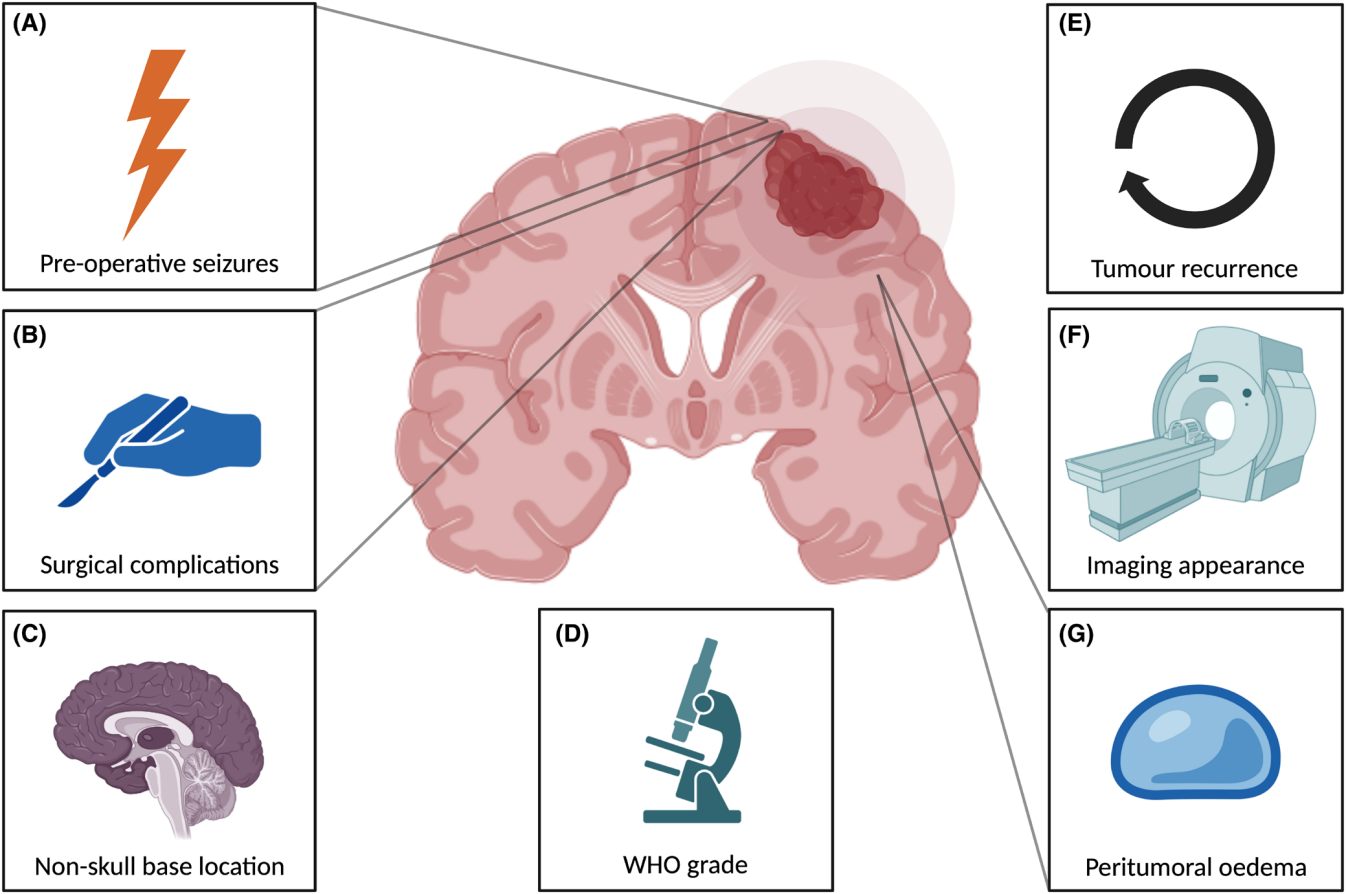
Summary of clinical associations with seizures in meningioma. (A) Preoperative seizures; (B) surgical complications; (C) non–skull‐base location; (D) World Health Organization (WHO) grade; (E) tumor recurrence; (F) imaging appearance; (G) peritumoral edema. Created with BioRender.com.

A meta‐analysis of studies reporting hazard ratios derived from multivariate regression analysis demonstrated that preoperative seizure history, non–skull‐base location, recurrence, and surgical variables such as postoperative complications are the strongest predictors of postoperative seizures^22^ (summarized in Figure 1); however, prospective studies are required to validate these findings. Major surgical complications including neurological infection, hydrocephalus, re‐craniotomy, ischemia, and symptomatic intracranial hemorrhage increase the risk of postoperative seizures.^20^, ^23^, ^24^ Although the mechanisms underlying these risk factors for seizures are probably varied, they likely have common final pathways of neuroinflammation, blood–brain barrier (BBB) disruption, and cortical irritation. Other clinic metrics that have been reported to help predict seizures, for example, Karnofsky Performance Score, do not reflect underlying tumor biology.^23^ Heterogenous contrast enhancement on imaging also seems to increase seizure risk.^23^ Higher WHO grade also increases the risk of postoperative seizures.^24^ People with non–skull‐base, usually parafalcine, parasagittal, or convexity meningioma, are at increased risk of postoperative seizures.^25^ For example, convexity meningioma are adjacent to epileptogenic neocortical gray matter, compared to skull‐base meningioma, which are not.^25^ From a surgical complication perspective, some convexity tumors are associated with the sagittal sinus or related large draining veins, and manipulation or sacrifice of these veins can cause venous engorgement and infarction, which increases the risk of seizures.^26^ Convexity meningioma are more frequently a higher WHO grade (compared to skull‐base meningioma), which although usually controlled for in regression analysis, are an independent risk factor for cortical invasion and related increased seizure risk.^27^, ^28^ Finally, recurrence may mediate an increased risk of postoperative seizures through reactivation of a previous epileptogenic lesion or generation of a new one.^20^, ^29^, ^30^ A related possible mechanism is that subtotal resection can be associated with a greater risk of postoperative seizures.^31^

Seizure prediction nomograms, such as STAMPE2, which stands for sensorimotor deficit, tumor progression, age, major surgical complications, preoperative epilepsy, epileptiform electroencephalography (EEG) activity, and edema,^20^ have integrated clinical features, EEG, and imaging appearances to develop seizure prediction nomograms, but this tool has not been externally validated in a prospective cohort.^32^ These types of tools do not increase our understanding of the mechanism of epileptogenesis in meningioma. Objective biofluid (including blood, urine, cerebrospinal fluid [CSF], tears), tumor tissue, imaging, or EEG‐based biomarkers would be more clinically useful adjuncts. Blood and CSF are the most extensively investigated biofluids in this context and will be discussed further.

## 3 MENINGIOMA BIOLOGY AND EVIDENCE FOR NEUROINFLAMMATION

Meningioma arise from the meningeal arachnoid cap cells,^33^ although dural border and arachnoid barrier cells have also been implicated.^34^, ^35^ Meningioma are a group of phenotypically and genetically diverse tumors with 15 histological subtypes.^4^ They are “extra‐axial” tumors, as they arise from the meninges, superficial to the brain itself, and displace the brain with increasing tumor volume. Meningioma generally retain a tissue plane between themselves and the brain, although they may become invasive and breach the pia mater and basement membrane.

Less is known about the meningioma tumor microenvironment compared to other central nervous system (CNS) tumors.^36^ Tumor‐associated microglia/macrophages (TAMs) are the most abundant infiltrating immune cells found in meningioma.^36^ TAMs are plastic and exist on a spectrum of polymerized states that is generally dichotomized into M1 (pro‐inflammatory/anti‐tumoral) and M2 (anti‐inflammatory/pro‐tumoral) phenotypes.^37^ Brain invasion by meningioma is required to elicit an immune response from the brain or blood,^36^ as a monocytic response at the brain–tumor interface is seen only in brain‐invasive meningioma.^38^ Immune cell infiltrate also consists of macrophages, CD4+ T cells of the Th1 and Th2 subtype, CD8^+^ cytotoxic T cells, mast cells, and some B cells.^39^ Mast cells are present in up to 90% of high‐grade meningioma, are found mainly in perivascular areas of tumor, and seem to correlate with peritumoral edema.^40^ They also reside on the brain side of the BBB and communicate with other cells.^41^ Meningioma are located outside the BBB,^42^ which is breached in invasive meningioma^43^; therefore the monocytic infiltration seen in meningioma probably comes from blood‐ or skull‐derived macrophages.^36^, ^44^

This diverse tumor microenvironment in meningioma contributes to a range of cytokines, chemokines, and growth factors with both pro‐ and anti‐inflammatory effects.^36^ Interleukin 3 (IL‐3), IL‐6, granulocyte colony‐stimulating factor (G‐CSF), granulocyte monocyte colony‐stimulating factor (GM‐CSF), interferon gamma (IFN‐γ), oncostatin M (OSM), leukemia inhibitor factor (LIF), tumor necrosis factor alpha (TNF‐α), TNF‐β, IL‐1α, IL‐1β, transforming growth factor (TGF) family, IL‐8, chemokine ligand 2/monocyte chemoattractant protein 1 (CCL2/MCP‐1), chemokine (C‐X‐C motif) ligand 1/2/3 (CXCL1/2/3), CXCL9, and CXCL10 have all been found expressed in meningioma.^36^ Certain cytokines such as G‐ and CM‐CSF have been found to correlate with WHO grade in meningioma.^45^ Some cytokines such as IL‐6, CXCL12, and TGF‐β are expressed in an autocrine manner within meningioma cells, which are otherwise involved in a signaling mechanism and tumor behavior.^46^, ^47^, ^48^, ^49^ Other cytokines expressed in meningioma such as IL‐1β, IL‐1α, and TNF‐α have not had corresponding receptors detected yet.^36^ Some paracrine cytokine signaling has also been demonstrated in endothelial cells and TAMs.^50^ It is not clear whether parenchymal microglia and astrocytes contribute to cytokine release in meningioma.^36^

Neuroinflammation is a CNS response to insults including tissue damage.^51^, ^52^ This inflammatory response consists of the synthesis and release of inflammatory molecules from brain‐resident activated microglia, astrocytes, neurons, and vasculature.^52^ Neuroinflammation can be a normal response to noxious stimuli in order to maintain homeostasis.^53^ Neuroinflammatory responses that are too intense or long in duration can cause harm to the CNS, as seen in conditions such as neurodegenerative disease,^54^ autoimmune encephalitis,^55^ and epilepsy.^56^ Increased neural activity such as seizures themselves can evoke an inflammatory response.^57^ Finally, surgery itself is an inflammatory, traumatic insult to the brain and surroundin tissues.^58^, ^59^

## 4 PROPOSED MECHANISMS OF EPILEPTOGENESIS IN BRAIN TUMORS

**FIGURE 2:**
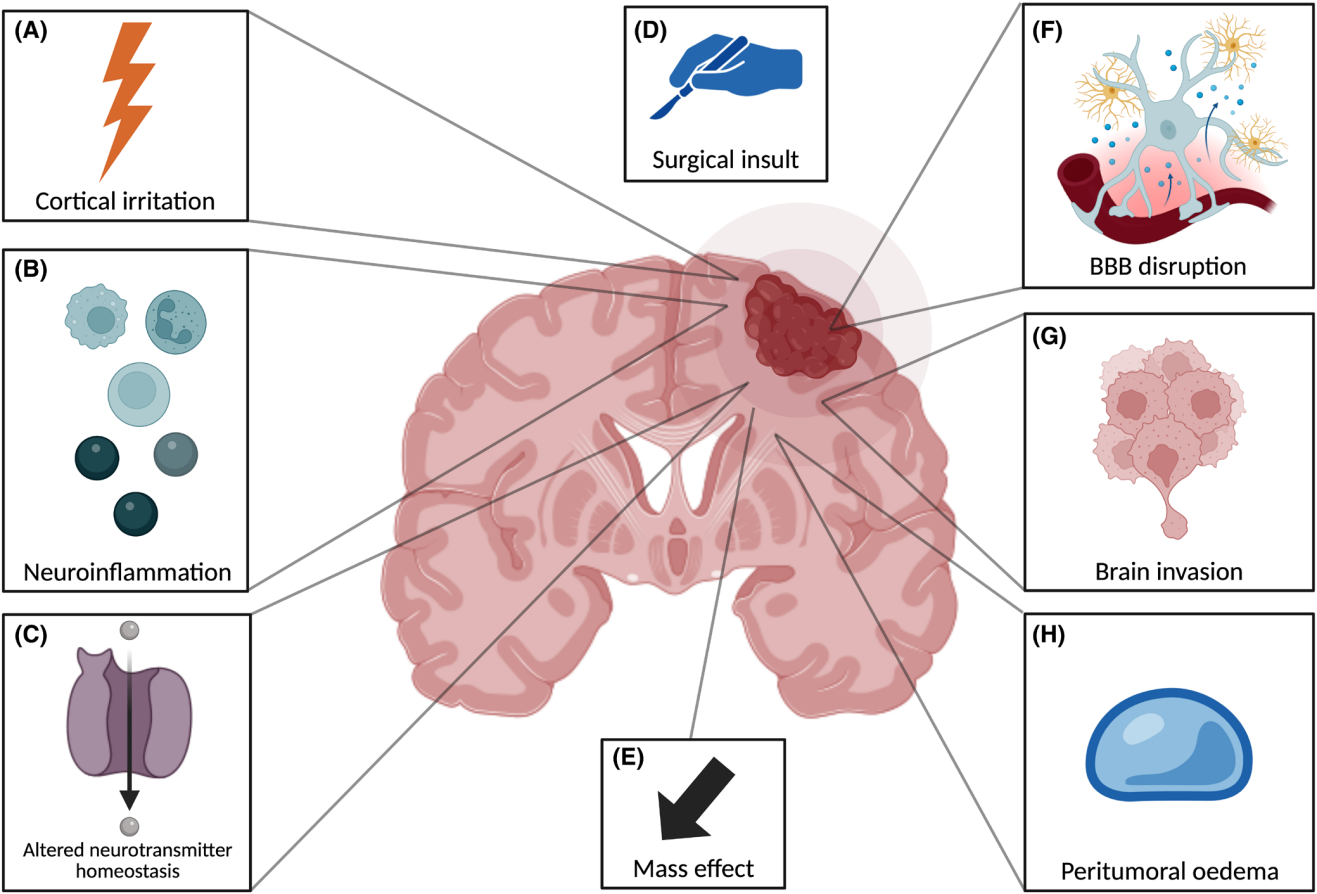
Summary of putative mechanisms of epileptogenesis in meningioma. (A) Cortical irritation; (B) neuroinflammation; (C) altered neurotransmitter homeostasis; (D) surgical insult; (E) mass effect; (F) blood–brain barrier disruption; (G) brain invasion; (H) peritumoral edema. Created with BioRender.com.

The mechanisms underpinning brain tumor–related seizures are most likely multifactorial and related to morphological, biochemical, and metabolic causes.^60^ Brain tumors likely cause cortical hyperexcitability due to the irritation caused by mass effect, brain invasion, and peritumoral brain edema.^61^, ^62^, ^63^, ^64^, ^65^ BBB disruption, altered neurotransmitter homeostasis, gap junction alterations, and altered interplay between excitatory and inhibitory neuronal populations also occur.^66^, ^67^, ^68^ These mechanisms are well‐summarized in multiple reviews and presented visually in Figure 2 as they pertain to meningioma; however, no single theory can fully explain the patterns of seizure incidence in all brain tumors.^66^, ^67^, ^69^

Epileptiform activity has been found to originate in the peritumoral border of human gliomas,^70^ or 1–2 mm away from the tumor mass in animal models where invading tumor cells surround neurons.^71^ Similar evidence does not yet exist for meningioma. One study included a single meningioma patient, although recordings were inconclusive,^70^ as only two epileptiform transients were identified on EEG and neither could be localized. In contrast, intrinsic tumors had between 6 and 42 epileptiform transients identified, many of which could be localized to a particular distance from the tumor border.^70^

The mechanisms of epileptogenesis vary for different tumor types as some are intra‐axial and infiltrate brain parenchyma, for example gliomas, whereas extra‐axial tumors such as meningioma generally distort and occasionally invade the brain.^66^ There are fundamental biological differences between intra‐axial and extra‐axial tumors, such as the glutamatergic synapses between neurons and glioblastoma cells,^72^ that do not exist in meningioma. There is little direct mechanistic evidence for the pathophysiology of seizures in meningioma. The putative mechanism is that seizures most likely arise due to peritumoral changes,^18^ although peritumoral tissue is infrequently obtained during meningioma surgery,^73^ which may have contributed to the paucity of evidence. Brain invasive meningioma, in a similar way to intra‐axial tumors, invade parenchyma as finger‐like projections and disrupt the pial‐glial basement membrane,^74^ leading to a change in astrocytes,^75^ which changes the tumor microenvironment and contributes to epileptogenesis. Brain invasion is also associated with, but not a prerequisite for, peritumoral brain edema.^76^, ^77^ Most meningioma patients (53%–90%) with preoperative seizures are cured of seizures following surgery,^5^ which suggests that the mechanical tumor burden may be most relevant for most patients. In patients with refractory seizures, additional mechanisms must be involved.

Peritumoral edema is a strong predictive variable for preoperative seizures in meningioma.^78^ Meningioma‐related edema is likely multifactorial, with possible mechanisms including vasogenic extravasation, venous stasis (interstitial), and direct compression (cellular).^79^ Peritumoral edema may be a surrogate marker for other mechanisms that drive epileptogenesis, such as tumor angiogenesis and an increase in pial blood supply.^80^ In meningioma, molecules such as vascular endothelial growth factor (VEGF),^81^ matrix metalloproteinase‐9 (MMP9),^82^ hypoxia‐inducible factor alpha (HIF‐1α),^83^ and IL‐6^84^ have been implicated in the development of peritumoral brain edema. This process may cause local hypoxia, neurotransmitter alterations, and BBB disruption, which are implicated in tumor‐related epileptogenesis.^64^ High levels of the excitatory neurotransmitter glutamate have also been found in vasogenic edema.^85^ Dexamethasone is commonly prescribed to patients with symptomatic peritumoral edema.^86^ At a transcriptional level, glucocorticoids suppress the synthesis of several cytokines and chemokines involved in regulating the inflammatory reaction,^87^ and also reduce the permeability of tumor capillaries in animal models.^88^, ^89^ Corticosteroids also appear to have an antiseizure effect in some seizure syndromes, for example, infantile spasms and electrical status epilepticus in sleep.^90^

## 5 PUTATIVE NEUROINFLAMMATORY MECHANISMS FOR MENINGIOMA‐ASSOCIATED SEIZURES

There is emerging evidence that inflammatory mediators are involved in epileptogenesis in animal models and human seizure syndromes.^90^, ^91^ IL‐1β is consistently elevated in the presence of seizures in a range of etiologies.^90^ High‐mobility group box‐1 (HMGB1) is a key initiator of neuroinflammation following epileptogenic injuries, and its inactivation contributes to seizure generation in animal models.^91^ Nevertheless, studies of inflammation in seizure syndromes often have a problem demonstrating causality.

In animal models, selective antagonism by the endogenous IL‐1 receptor antagonist (IL‐1ra) can reduce seizure frequency after traumatic brain injury.^92^ Neuroinflammatory markers such as macrophages and the proinflammatory cytokines IL‐6 and TNF‐α cause loss of potassium and neurotransmitter glutamate homeostasis,^93^ directly affecting neurovascular and glial function, and thereby increasing the risk of seizures. There is evidence that immune cells promote epileptogenesis through mediating BBB breakdown.^94^ Recruitment of neutrophils and macrophages following exposure to foreign organism, in this case a virus, disrupts the BBB, due to endothelial dysfunction caused by cytokines.^94^, ^95^ Finally, cytokines themselves can also cause BBB failure.^96^

Few studies have investigated the role of inflammation in meningioma.^97^, ^98^ In a study of 707 patients with meningioma, the relationship between seizure burden and low‐dose aspirin use (in 107 patients) was investigated. The study hypothesis was the possible association between MIB‐1 index (a measure of tumor proliferation) and cyclooxygenase 2 (COX‐2) expression.^98^ Aspirin therapy was associated with lower MIB‐1 index, in a subset of patients (women ≥60 years old) and there were more WHO Grade 2 meningiomas in the no‐aspirin group (33% vs 15% in the aspirin group), which was not statistically significant. Aspirin was also associated with a reduced burden of symptomatic epilepsy at presentation in non–skull‐base meningioma in both genders but WHO grade was not included in the multivariate analysis. The study authors concluded that anti‐inflammatory therapy may modify the inflammatory tumor microenvironment; however, they did not collect sufficient mechanistic evidence to support that conclusion, and only measured C‐reactive protein and fibrinogen, which were not different between groups, and the duration of aspirin therapy for different patients was not collected or reported. Although these individual studies provide snapshots from different angles of how inflammation may be involved in the generation or propagation of seizures, proving causality is difficult. Taken together, the evidence points toward inflammation being involved in meningioma‐associated seizures but requires further pre‐clinical mechanistic study to prove. Nevertheless, predicting seizure risk in meningioma is of great value to patients and clinicians and can proceed even while pre‐clinical mechanistic work continues in parallel.

## 6 POTENTIAL NEUROINFLAMMATORY BIOMARKERS OF EPILEPTOGENESIS IN MENINGIOMA

Blood biomarkers of neuroinflammation for predicting seizure risk in meningioma hold promise due to the theories of epileptogenesis presented above and findings in other seizure syndromes.^99^ However, there is no convincing evidence for them in symptomatic epilepsy of any cause.^100^

A retrospective study of 97 patients undergoing meningioma resection evaluated commonly measured blood inflammatory markers (white blood cell count, neutrophils, lymphocytes, monocytes, platelets, neutrophil‐lymphocyte ratio, and derived neutrophil‐lymphocyte ratio) before and after surgery and followed patients for 1 year.^97^ Patients with postoperative de novo seizures had a significantly higher white blood cell count than those who never had a seizure, but this finding was poorly predictive of de novo seizures at 1 year (area under the curve [AUC] 0.61). Derived neutrophil‐lymphocyte ratio was more predictive of seizures at 1 year (AUC 0.83). Although this study was careful to exclude patients with inflammatory conditions, perioperative infection, other medical illness, or operative complication, the study authors acknowledged that patient stress or undetected viral illness may have influenced the measured inflammatory markers. These limitations also apply to measuring small inflammatory molecules such as cytokines, the levels of which are known to vary with other variables such as circadian rhythm and exercise.^101^ It was unclear how many patients in each group were given perioperative dexamethasone, which can affect inflammatory markers.^87^ Full blood counts are cheap and used routinely in the clinical environment, but they do not shed light on the mechanistic role of inflammation in epileptogenesis. Consequenly, measurement of inflammatory cytokines may be more informative.

Inflammatory cytokines and chemokines have been evaluated as prognostic markers in seizure syndromes, but their short half‐lives and low concentration in plasma (e.g., IL‐1β half‐life 2.5 h intracellularly^102^), have hampered their evaluation and produced discordant results.^51^ Certain cytokines involved in the inflammatory cascade are more stable and exist in higher concentrations, such as IL‐6, which can be more easily measured.^103^ Inflammatory molecules measurable in blood should ideally be CNS‐specific and analysis should not be confounded by non‐specific release from peripheral sources, for example, complement factors that are also released from the liver. The list of possible cytokines that can be measured is extensive.^90^ Those most investigated with respect to seizures and meningioma include IL‐1ra, IL‐1β, IL‐6, IL‐10, IFN‐γ, and TNF‐α. Most are present in blood, and some are also in the CSF.

In a meta‐analysis of 51 different inflammatory mediators in brain tissue, CSF, and blood serum of patients with epilepsy,^90^ levels were compared between epilepsy patients and controls, and in a few studies, between post‐ictal and baseline levels (Table 1). Peripheral blood mononuclear cell (PBMC) stimulation studies in patients with epilepsy demonstrated a hyper‐reactive immune state with increased IL‐1 family cytokines, IL‐6, IL‐10, and IFN‐α compared to controls.^90^

**TABLE 1:** Summary of inflammatory mediators measured in blood, CSF, and brain tissue from epilepsy patients, adapted from de Vries.^90^

Inflammatory mediator	Blood	CSF	Brain tissue
IL‐1α	↑		↑
IL‐1β	↑	↑	↑
IL‐1ra	↑	↑	↑
IL‐6	↑	↑	↑
IL‐7			↑
CXCL8/IL‐8	↑	↑	↑
IL‐10		↑	
IL‐12		↑	
IL‐13		↑	↑
IL‐17	↑		
IFN‐α	↑		
IFN‐γ	↑	↑	
TNF‐α	↑	↑	
CCL2‐5			↑
CCL4	↑		
CCL11	↑		
CCL19			↑
CCL22			↑
CXCL10	↑		
CX3CL1	↑	↑	
HMGB1	↑		
bFGF	↑		

Abbreviation: bFGF, basic fibroblast growth factor; CCL, chemokine ligand; CXCL, chemokine (C‐X‐C motif) ligand; CX3CL1, C‐X3‐C motif chemokine ligand 1; HMGB1, high‐mobility group box 1; IFN, interferon; IL, interleukin; TNF, tumor necrosis factor.

In temporal lobe epilepsy specifically, IL‐1α, IL‐1β, CCL3, CCL4, and CCL5 levels were found to be higher in brain tissue than in controls.^90^ In serum, IL‐1ra levels were no different between patients and controls,^104^, ^105^ were lower in one study,^106^ IL‐1β was unchanged,^104^, ^105^ the IL‐1ra/IL‐1β ratio was lower after a seizure,^106^ and IL‐6 was elevated.^90^ Meta‐analysis of studies measuring IL‐1β and IL‐6 in febrile seizures found no increased expression in CSF or serum,^90^ despite at least one individual study finding increases.^107^ One study found an increase in IL‐1ra/IL‐1β ratio.^108^ Studies of inflammatory mediators in other seizure disorders have found increased IL‐6 expression in resected tissue in focal cortical dysplasia and tuberous sclerosis.^109^ IL‐1β has been found to be highly upregulated in focal cortical dysplasia and glioneuronal tumors.^110^ The different findings between brain tissue, CSF, and blood serum could reflect compartmentalized components of the immune system, especially as numerous chemokines such as the CCL family and CX3CL1 may be involved in focal brain inflammation, whereas IL‐6 is more of a general marker of systemic inflammation.^90^ One study of drug‐resistant epilepsy found that the plasma ratio between CCL17 and sICAM5 was predictive of seizure activity.^111^ The short half‐lives of cytokines makes sampling them from different body fluids and tissues and expecting similar results problematic. Elective meningioma surgery provides an excellent opportunity to assess inflammatory mediators as potential predictors of postoperative seizures.

Few studies have measured inflammatory mediators in the blood plasma of patients with meningioma,^112^, ^113^, ^114^, ^115^ although others have evaluated the tumor itself for interleukin receptors.^116^, ^117^, ^118^, ^119^ Most studies have only measured IL‐6 in relation to discharge outcome or survival following discharge,^112^ based on the association with proliferation rate.^47^ There is a clear need to measure these molecules with high fidelity in meningioma populations and given the elective meningioma setting, samples could be taken before, during, and after surgery in patients with and without seizures, while also following them over time for seizure activity.

## 7 CURRENT CHALLENGES

Despite some advances in the understanding of epileptogenesis in meningioma and potential seizure biomarkers described in this review, much of the former is extrapolated from infiltrative gliomas and much of the latter has been extrapolated from either epilepsy syndromes or preclinical animal models of trauma, status epilepticus, or other seizure models. This limits the extrapolation of the findings to patients with meningioma and seizures. Meningiomas are a group of phenotypically and genetically diverse tumors,^120^ and there are likely to be both common epileptogenic mechanisms as well as specific changes related to meningioma genetics. Whether a single unifying predictive biomarker exists is not known.

A recurring problem with biomarker discovery in seizure syndromes is that many of the potential biomarkers cannot be proved to cause or predict epilepsy, as they are collected from people who already have epilepsy. Nevertheless, these biomarkers may be useful in understanding the mechanisms that are maintaining a susceptibility to seizures. In the elective meningioma setting, investigators can collect samples from the two‐thirds of patients with seizure‐naïve meningioma before some of them develop seizures—this would represent a unique cohort of patients to further our understanding of epileptogenesis.

Finally, any promising biomarker must be convenient and cost‐effective for clinical services to collect and interpret. Biomarkers identified in the bloodstream would be readily accessible but must be sufficiently chemically stable to withstand transport and analysis by a clinical laboratory, which may limit the utility of some molecules with short half‐lives such as some inflammatory mediators.

## 8 CONCLUSIONS AND FUTURE DIRECTIONS

The mechanisms behind epileptogenesis in symptomatic epilepsy and in brain tumors are not known. Most of the current knowledge is derived from infiltrative glioma and animal models. Meningioma is a potential model for investigating the mechanisms behind developing focal epilepsies, and these mechanisms may also be relevant to other types of epilepsy. Investigating blood biomarkers (including inflammatory and genetic) serves the dual purpose of improving seizure prognostication for patients, identifying those most likely to benefit from treatment with ASMs, as well as shedding light on inflammatory and genetic pathways of epileptogenesis. Ultimately, this might help us develop actual seizure prevention treatments.

## FUNDING INFORMATION

This study did not receive any funding or financial support.

## CONFLICT OF INTEREST STATEMENT

M.D.J. has received honoraria from Servier, Integra Health, Novocure, myTomorrows, and GlaxoSmithKlein. The remaining authors have no conflicts of interest. We confirm that we have read the Journal's position on issues involved in ethical publication and affirm that this report is consistent with those guidelines.

## Data Availability

Data sharing is not applicable to this article as no new data were created or analyzed in this study.
